# Prevalence and risk factors of intrahepatic cholestasis of pregnancy in a Chinese population

**DOI:** 10.1038/s41598-020-73378-5

**Published:** 2020-10-01

**Authors:** Xing-Xing Gao, Meng-Ying Ye, Yan Liu, Jin-Yan Li, Li Li, Wei Chen, Xue Lu, Guiying Nie, Yuan-Hua Chen

**Affiliations:** 1 Department of Histology and Embryology, Anhui Medical University, No. 81 Meishan Road, Shushan District, Hefei, 230032 Anhui People’s Republic of China; 2 Department of Obstetrics and Gynecology, First Affiliated Hospital of Anhui Medical University, Hefei, 230022 People’s Republic of China; 3 Key Laboratory of Environmental Toxicology of Anhui Higher Education Institutes, Anhui Medical University, Hefei, 230032 People’s Republic of China; 4 Implantation and Placental Development Laboratory, RMIT University, Bundoora, VIC 3083 Australia; 5 Implantation and Placental Development Laboratory, Centre for Reproductive Health, Hudson Institute of Medical Research, Melbourne, 3168 Australia

**Keywords:** Diseases, Medical research

## Abstract

Studies on the risk factors for intrahepatic cholestasis of pregnancy (ICP) in a population-based cohort are lacking. We assess the prevalence and risk factors of ICP in a Chinese population. In this study, a cohort study was conducted that included 12,200 eligible pregnant women. The overall incidence of ICP in this cohort was 6.06%. With increasing maternal age, the incidence of ICP decreased in women younger than 30 years of age but increased in those older than 30. With increasing pre-pregnancy BMI, the incidence of ICP decreased if the pre-pregnancy BMI was less than 23 kg/m^2^ but increased if it was 23 kg/m^2^ or higher. Further analysis showed that the risk of ICP increased when maternal age was < 25 years (Adjusted RR  2.01; 95% CI 1.64–2.47) or ≥ 35 years (Adjusted RR  1.34; 95% CI 1.02–1.76). Furthermore, an increased risk of ICP was associated with pre-pregnancy underweight (adjusted RR  1.27; 95% CI 1.04–1.56), inadequate gestational weight gain (GWG) (adjusted RR  1.58; 95% CI 1.28–1.96), lower maternal education (adjusted RR  2.96; 95% CI 2.35–3.74), multiparity (adjusted RR  1.54; 95% CI 1.23–1.93), and twin/multiple pregnancies (adjusted RR  2.12; 95% CI 1.25–3.58). Maternal age (< 25 or ≥ 35 years), underweight, inadequate GWG, lower maternal education, multiparity, and twin/multiple pregnancies were identified as risk factors of ICP.

## Introduction

Intrahepatic cholestasis of pregnancy (ICP), also known as obstetric cholestasis, is defined as the presence of pruritus in combination with a total serum bile acid (TBA) level above 10 μmol/L during the second and third trimesters of pregnancy^1,2^. ICP is a pregnancy-specific liver disease, and the incidence varies from 0.1 to 15.6% depending on geography and ethnicity^3^. ICP is more common in South Asia, South America and Scandinavia. The etiology of ICP is multifactorial, and may be associated with increased estrogen levels as well as altered expression of hepatobiliary transport proteins during pregnancy^4,5^. ICP increases the risk of adverse fetal outcomes. Several epidemiological studies have shown that ICP is associated with spontaneous and iatrogenic preterm delivery^6–8^. Several experimental studies have also demonstrated that ICP is a leading cause of stillbirth and neonatal demise^9–11^. Furthermore, many studies show an association between ICP and respiratory distress syndrome, fetal intrauterine growth restriction, a low (< 7) 5-min Apgar score, and meconium-stained fluid^12–14^. A recent randomised control trial in pregnant women with ICP reports that treatment with ursodeoxycholic acid, a common agent for treating ICP, not only does not significantly reduces serum TBA levels and improves pruritus and liver functions, but also does not decrease the occurrence of adverse fetal outcomes^15^.

ICP is also associated with increased risks of adverse maternal outcomes. Women who have experienced ICP have increased risks of later-life cardiovascular diseases, autoimmune-mediated conditions, diabetes mellitus, hepatobiliary diseases and carcinoma^16,17^. Epidemiological studies also report that women with ICP are at increased risks for gestational diabetes mellitus, dyslipidemia and pre-eclampsia^18–20^.

To date, most of the studies have focused on the association between ICP and adverse fetal and maternal outcomes. Although the incidence of ICP differs significantly among various countries and ethnicities^21,22^, studies on the risk factors for ICP in a population-based cohort are lacking.

The objective of the current study was to assess the prevalence and risk factors of ICP in a Chinese population. We found that pregnancy at a young or advanced maternal age, underweight, inadequate GWG, lower maternal education, multiparity, and twins/multiple pregnancies were associated with an increased risk of ICP.

## Results

### The demographic characteristics and laboratory measurements of participants

The incidence of ICP in this cohort was 6.06% (739/12,200, Table 1). The demographic characteristics of study population are summarized in Table 1. There were significant differences in maternal age, maternal pre-pregnancy BMI, gestational weight gain, maternal education, mode of delivery and parity between control and ICP groups. The mean gestational age was significantly lower in those with ICP as compared with controls (Table 1). There was also a significant difference in the prevalence of twins or multiplets between the two groups (Table 1). However, gravidity and gestational diabetes were not significantly different between the two groups (Table 1). Furthermore, the levels of serum TBA, aspartate transaminase, alanine transaminase, total bilirubin, direct bilirubin and indirect bilirubin were all significantly higher in those with ICP than controls (Table 2).

**Table 1:** Demographic characteristics of the study population.

Demographic variables	Control (n = 11,461)	ICP (n = 739)	*p* value
**Maternal age (years)**			
< 25 [n (%)]	1714 (15.0)	174 (23.5)	< 0.001
25–34 [n (%)]	8437 (73.6)	463 (62.7)	
≥ 35 [n (%)]	1310 (11.4)	102 (13.8)	
**Maternal BMI [n (%)]**			
Underweight (< 18.5 kg/m^2^)	1927 (16.8)	149 (20.2)	0.010
Normal weight (18.5 ≤ BMI < 25.0 kg/m^2^)	8507 (74.2)	510 (69.0)	
Overweight (25.0 ≤ BMI < 30 kg/m^2^)	872 (7.6)	64 (8.7)	
Obesity (≥ 30 kg/m^2^)	155 (1.4)	16 (2.2)	
**Gestational weight gain [n (%)]**^**a**^			
Inadequate	1531 (13.4)	180 (24.4)	< 0.001
Adequate	3790 (33.1)	251 (34.0)	
Excessive	4618 (40.3)	224 (30.3)	
Data missing	1522 (13.3)	84 (11.4)	
**Maternal education [n (%)]**^**b**^			
Low	3660 (31.9)	368 (49.8)	< 0.001
Medium	3618 (31.6)	199 (26.9)	
High	3770 (32.9)	146 (19.8)	
Data missing	413 (3.6)	26 (3.5)	
**Mode of delivery [n (%)]**			
Vaginal	5042 (44.0)	292 (39.5)	0.009
Cesarean	6419 (56.0)	447 (60.5)	
**Parity [n (%)]**			
Primiparous	8388 (73.2)	477 (64.5)	0.001
Multiparous	2819 (24.6)	214 (29.0)	
Data missing	254 (2.2)	48 (6.5)	
**Gravidity [n (%)]**			
Primigravid	5946 (51.9)	352 (47.6)	0.159
Multigravid	5279 (46.1)	339 (45.9)	
Data missing	236 (2.0)	48 (6.5)	
**Gestational diabetes [n (%)]**			
No	10,496 (91.58)	667 (90.26)	0.127
Yes	965 (8.42)	72 (9.74)	
Gestational age (weeks, mean ± SD)	38.7 ± 2.6	37.5 ± 2.8	< 0.001
**Twin or multiple pregnancies**			
No	11,304 (98.6)	717 (97.0)	0.001
Yes	157 (1.4)	22 (3.0)	

The differences between the two groups were compared using Chi-square test (χ^2^ test).

^a^Inadequate: gestational weight gain (GWG) < 12.5 kg in underweight women, < 11.5 kg in normal-weight women, < 7 kg in overweight women, and < 5 kg in obese women. Adequate: 12.5 ≤ GWG ≤ 18 kg in underweight women, 11.5 ≤ GWG ≤ 16 kg in normal-weight women, 7 ≤ GWG ≤ 11.5 kg in overweight women, and 5 ≤ GWG ≤ 9 kg in obese women. Excessive: GWG > 18 kg in underweight women, > 16 kg in normal-weight women, > 11.5 kg in overweight women, and > 9 kg in obese women.

^b^Low, junior school or less; Medium, high school graduate or equivalent; High, College or above.

**Table 2:** Laboratory measurements within the study population.

Laboratory measurements	Control (n = 11,461)	ICP (n = 739)	*p* value
TBA (μmol/L)	2.90 (2.40)	16.54 (17.80)	< 0.001
Alanine transaminase (U/L)	29 (18)	66 (149)	< 0.001
Aspartate transaminase (U/L)	18 (9)	58 (110)	< 0.001
Total bilirubin (μmol/L)	7.45 (3.93)	10.01 (8.00)	< 0.001
Direct bilirubin (μmol/L)	1.55 (0.91)	2.82 (3.92)	< 0.001
Indirect bilirubin (μmol/L)	6.08 (2.05)	6.68 (3.79)	< 0.001

Date were median (IQR) for nonnormally distributed parameters.

The differences were analyzed using non-parametric statistics (Mann–Whitney U test).

### Association between demographic characteristics as a categorical variable and the risk of ICP

The association between demographic characteristics and the risk of ICP was analyzed (Table 3). Compared to a maternal age range of 25–34 years, younger than 25 or older than 35 was associated with an increased risk of ICP, with an adjusted RR of 2.01 (95% CI 1.64–2.47) and 1.34 (95% CI 1.02–1.76) respectively (Table 3). Referring to pre-pregnancy body weight, underweight was associated with an increased risk of ICP (adjusted RR 1.27; 95% CI 1.04–1.56); however no significant associations were observed between overweight, obesity and the risk of ICP (Table 3). When gestational weight gain (GWG) and the risk of ICP was analyzed, inadequate GWG was associated with an increased risk of ICP (adjusted RR 1.58; 95% CI 1.28–1.96), whereas excessive GWG decreased the risk of ICP (adjusted RR 0.72; 95% CI 0.62–0.92). The association between maternal education and the risk of ICP was also analyzed. Compared to those with high education, mothers with medium and low education had a higher risk of ICP, and adjusted RRs were 1.40 (95% CI 1.10–1.78) and 2.96 (95% CI 2.35–3.74) respectively (Table 3). When parity and gravidity were considered, multiparity increased the risk of ICP compared to primiparity (adjusted RR 1.54; 95% CI 1.23–1.93), but gravidity was not significantly associated with ICP (Table 3). Furthermore, twin or multiple pregnancies were associated with a 2.12 fold increase (adjusted 95% CI 1.25–3.58) of ICP (Table 3).

**Table 3:** Association between demographic characteristics as a categorical variable and ICP based on multiple logistic regression analyses.

Parameters	Crude models		Adjusted models	
	OR (95% CI)	*p*	OR (95% CI)	*p*
**Maternal age (years)**^**a**^				
< 25	1.85 (1.54, 2.22)	< 0.001	2.01 (1.64, 2.47)	< 0.001
25–34	1.00		1.00	
≥ 35	1.42 (1.14, 1.77)	0.002	1.34 (1.02, 1.76)	0.035
**Maternal BMI**^**b**^				
Underweight	1.29 (1.07, 1.56)	0.008	1.27 (1.04, 1.56)	0.021
Normal weight	1.00		1.00	
Overweight	1.22 (0.94, 1.60)	0.141	1.13 (0.85, 1.51)	0.411
Obesity	1.72 (1.02, 2.90)	0.041	1.68 (0.99, 2.86)	0.056
**Gestational weight gain**^**b**^				
Inadequate [n (%)]	1.78 (1.45, 2.17)	0.001	1.58 (1.28, 1.96)	< 0.001
Adequate [n (%)]	1.00		1.00	
Excessive [n (%)]	0.70 (0.61, 0.88)	< 0.001	0.72 (0.62, 0.92)	0.006
**Maternal education**^**c,d**^				
Low	2.60 (2.13, 3.16)	< 0.001	2.96 (2.35, 3.74)	< 0.001
Medium	1.42 (1.14, 1.77)	0.002	1.40 (1.10, 1.78)	0.006
High	1.00		1.00	
**Parity**^**e**^				
Primiparous	1.00		1.00	
Multiparous	1.34 (1.13, 1.59)	0.001	1.54 (1.23, 1.93)	< 0.001
**Gravidity**^**f**^				
Primigravid	1.00		1.00	
Multigravid	0.92 (0.79, 1.08)	0.298	0.92 (0.74, 1.13)	0.408
**Gestational diabetes**^**g**^				
No	1.00		1.00	
Yes	0.86 (0.66, 1.10)	0.226	0.83 (0.62, 1.11)	0.213
**Twin or multiple pregnancies**^**g**^				
No	1.00		1.00	
Yes	2.21 (1.41, 3.47)	0.001	2.12 (1.25, 3.58)	0.005

^a^Adjusted for maternal BMI, gestational weight gain, maternal education, parity and gravidity.

^b^Adjusted for maternal age, maternal education, parity and gravidity.

^c^Low, junior school or less; Medium, high school; High, College or above.

^d^Adjusted for maternal age, maternal BMI, gestational weight gain, parity and gravidity.

^e^Adjusted for maternal age, maternal BMI, gestational weight gain, maternal education and gravidity.

^f^Adjusted for maternal age, maternal BMI, gestational weight gain, maternal education and parity.

^g^Adjusted for maternal age, maternal BMI, gestational weight gain, maternal education, parity and gravidity.

### Association between demographic characteristics as a continuous variable and the risk of ICP

**Figure 1:**
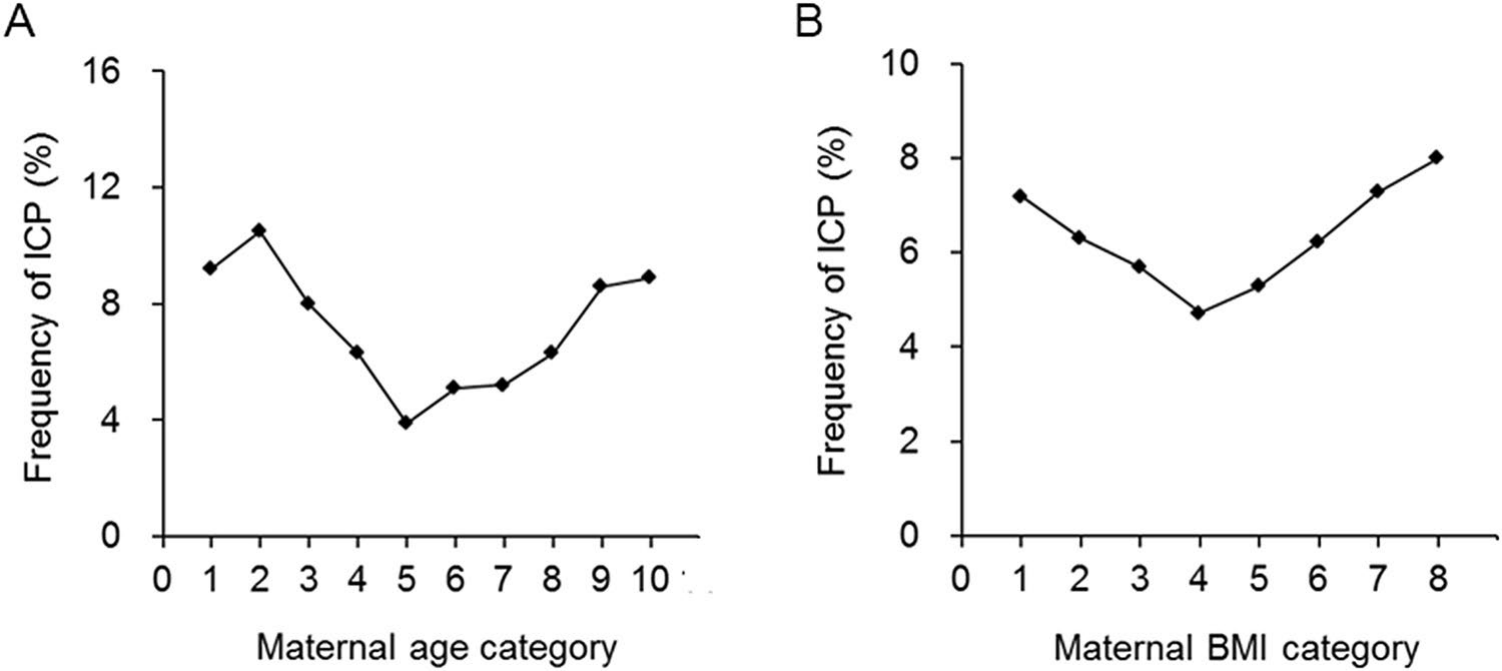
The relationship between ICP frequency and maternal age and BMI. (**A**) Maternal age in 10 categories. Age range in category 1, younger than 20.0 years of age (n = 98); 2, 20.0–22.4 (n = 617); 3, 22.5–24.9 (n = 1170); 4, 25–27.4 (n = 3117); 5, 27.5–29.9 (n = 2523); 6, 30.0–32.4 (n = 2451); 7, 32.5–34.9 (n = 809); 8, 35.0–37.4 (n = 821); 9, 37.5–39.9 (n = 279); 10, 40 or older (n = 315). (**B**) Maternal BMI in 8 categories. BMI range in category 1, less than 18.5 kg/m^2^ (n = 2074); 2, 18.5–19.9 (n = 2718); 3, 20.0–21.4 (n = 2994); 4, 21.5–22.9 (n = 1836); 5, 23.0–24.4 (n = 1189); 6, 24.5–25.9 (n = 673); 7, 26.0–27.4 (n = 329); 8, 27.5 or higher (n = 387).

Participants were divided into ten categories according to their pre-pregnancy age, and the incidence of ICP across these age ranges was analyzed (Fig. 1A). The lowest ICP was in those with a maternal age of 27.5–29.9 years (category 5, Fig. 1A). When the maternal age was less than 30 years (categories 1–5, Fig. 1A), increases in age was associated with an decreased risk of ICP. However, when the maternal age was 30 years or higher (categories 6–10, Fig. 1A), increases in age increased the incidence of ICP increased. We further analyzed the association between maternal age as a continuous variable and the risk of ICP. The adjusted RRs for an increase in maternal age of 1 SD were respectively 0.92 (95% CI 0.84–1.02) when all participants were analyzed as a cohort, 0.52 (95% CI 0.44–0.63) for those aged younger than 30 years, and 1.23 (95% CI 1.02, 1.48) for those aged 30 or older (Table 4).

**Table 4:** Association between demographic characteristics as a continuous variable and ICP based on multiple logistic regression analyses.

Parameters	98 ytrsCrude models		Adjusted models	
	OR (95% CI)^a^	*p*	OR (95% CI)^a^	*p*
**Maternal age categories**^**b**^				
All	0.91 (0.84, 0.98)	0.013	0.92 (0.84, 1.02)	0.104
< 30 years	0.54 (0.46, 0.64)	< 0.001	0.52 (0.44, 0.63)	< 0.001
≥ 30 years	1.28 (1.10, 1.49)	0.001	1.23 (1.02, 1.48)	0.035
**Maternal BMI categories**^**c**^				
All	0.99 (0.92, 1.07)	0.830	0.97 (0.90, 1.06)	0.501
< 23.0 kg/m^2^	0.79 (0.68, 0.91)	0.001	0.81 (0.69, 0.94)	0.007
≥ 23.0 kg/m^2^	1.23 (1.06, 1.41)	0.006	1.22 (1.05, 1.42)	0.012
Geatational weight gain^c^	0.69 (0.63, 0.78)	< 0.001	0.73 (0.67, 0.80)	< 0.001

^a^ORs were for an increase in covariates of 1 SD.

^b^Adjusted for maternal BMI, gestational weight gain, maternal education, parity and gravidity.

^c^Adjusted for maternal age, maternal education, parity and gravidity.

Participants were also divided into eight categories according to their pre-pregnancy BMI, and the incidence of ICP across these BMI ranges is shown in Fig. 1B. The incidence of ICP was lowest when the pre-pregnancy BMI was between 21.5 and 22.9 kg/m^2^) (category 4, Fig. 1B). When BMI was less than 23 kg/m^2^ (categories 1–4), its increase decreased the incidence of ICP (Fig. 1B). However, when the pre-pregnancy BMI was 23 kg/m^2^ or higher (categories 5–8), its increase increased ICP (Fig. 1B). We further analyzed the association between maternal BMI as a continuous variable and the risk of ICP. Adjusted RRs for an increase in maternal BMI of 1 SD were respectively 0.97 (95% CI 0.90–1.06) among all mothers, 0.81 (95% CI 0.69–0.94) for those with a pre-pregnancy BMI less than 23 kg/m^2^, and 1.22 (95% CI 1.05–1.42) when the pre-pregnancy BMI was 23 kg/m^2^ or higher (Table 4). Additionally, we investigated the relationship between GWG as a continuous variable and the risk of ICP, and the adjusted RR for an increase in GWG of 1 SD was 0.73 (95% CI 0.67–0.80) (Table 4).

## Discussion

To date, most of the studies have focused on the association between ICP and adverse fetal and maternal outcomes^6–8,23^. Although the differences including in maternal age and prepregnancy BMI were observed between ICP cases and controls^23^, studies on the risk factors for ICP in a Chinese population are lacking. The current study investigated the prevalence and risk factors of ICP in a Chinese population. Within 12,200 deliveries included in the study, 6.06% of the participants developed ICP. Increases in maternal age decreased the incidence of ICP when the maternal age was less than 30 years but increased it if the maternal age was 30 years or older. With increasing pre-pregnancy BMI, the incidence of ICP decreased when BMI was less than 23 kg/m^2^ but increased when the BMI was 23 kg/m^2^ or higher. Logistic regression models showed that in this cohort maternal age below 25 or above 35 years, pre-pregnancy underweight, inadequate GWG, lower maternal education, multiparity, and twins/multiple pregnancies were risk factors of ICP.

The incidence of 6.06% ICP found in the current study was comparable with findings of other cities in China^24,25^, but it was higher than that reported in the neighboring countries like Punjab Pakistan^26^. The disease was more common in South America, especially in Chile, where early study reported a 15.1% overall incidence and 24.1% among women of Araucanian Indian descent^27^. ICP was less common in North America, Central and Western Europe, which has been stable for many^28,29^. These variations in ICP prevalence might be due to differences in eating habit and nutritional status, geographic location, levels of health services, and differing diagnostic criteria. In addition, the overall incidence of ICP in a primarily Latina Los Angeles population was reported to be 5.6%, which is more than ten times higher than the previously reported prevalence within the United States and suggests an potential association between ICP and ethnicity^30^. Our current study found a complex association between pre-pregnancy BMI and ICP risks, which may provide an additional explanation to why the incidence of ICP differs among different populations and ethnicities.

Numerous reports agree that a suboptimal maternal age is linked to an increased risk of adverse pregnancy outcomes, such as preeclampsia, cesarean section, miscarriage, preterm delivery, fetal growth restriction, and neonatal mortality^31–33^. However, the association between maternal age and the occurrence of ICP remains unclear. In this cohort, 11.6% (1412/12,200) of deliveries were from women older than 35 years of age. The prevalence of advanced maternal age in our current cohort was lower than that of the European countries in 2016^34^, but was higher than that of China in 2009^35^. In addition, 15.5% (1888/12,200) of deliveries in this cohort were from women younger than 25 years of age, which was comparable with the overall rate of China in 2009^35^. Furthermore, this study found that an advanced as well as young maternal age, after adjustment for other maternal characteristics, was associated with an increased risk of ICP. Specifically, an older maternal age decreased ICP if the woman was younger than 30 years but increased it if she was older than 30. Our studies suggest that a maternal age between 27.5 and 32.5 is most optimal in lowering the risk of ICP.

This study found that twin/multiple pregnancies were associated with an increased risk of ICP. This may be related to higher levels of hormones such as estrogen and progesterone in these pregnancies^4,21,36^. Estrogen has also been demonstrated to induce cholestasis during pregnancy by inhibiting the expression of hepatic biliary proteins in rodents^37^. Previous studies have also demonstrated that progesterone metabolites could alter hepatic bile acid homeostasis by impairing the function of the major hepatic bile acid receptors^38^. Epidemiological studies show that the increased estrogen levels in twin pregnancies are associated with a greater risk of ICP^39^. Further experimental studies have proven that estrogen could inhibit the utilization of blood sugar, fat decomposition and free fatty acid release, while high free fatty acids induce liver injury and aggravate cholestasis^40^.

A 12-year population-based cohort in Sweden showed that women with ICP had an increased risk of gestational diabetes compared with normal pregnant women^23^. However, our study showed that there was no significant difference in gestational diabetes between the two groups. Actually, a previous study indicated that the incidence of gestational diabetes was significantly higher in Caucasian population but not in Asian population^18^, suggesting an ethnic disparity on the relationship between ICP and gestational diabetes.

An advantage of the current study is that it included a large population-based birth cohort and had adequate power to estimate associations using multivariable analyses. However, the study had three limitations. Firstly, as the cohort included only Chinese population, cautions are needed when the findings are branched out to other ethnic populations. Secondly, as the study was limited to only one hospital, a potential selection bias might not be completely excluded. A third limitation was the lack of information on the history of ICP in participants and their immediate family members, which prevented analyses of genetic susceptibilities to ICP^41,42^.

In summary, the present study analyzed the prevalence and risk factors of ICP in a Chinese population that included 12,200 eligible pregnant women. In this cohort, the overall incidence of ICP was 6.06%, and the risk factors of ICP were maternal age below 25 or above 35 years, pre-pregnancy underweight, inadequate GWG, lower maternal education, multiparity and twin/multiple pregnancies. Our studies provide new understandings of ICP, which may facilitate the prioritization of medical interventions, resource assignments and policy making. In particular, our results may aid the prediction of pregnancies with a high risk of ICP, providing clinicians with time to plan and strategize their patients’ maternal/fetal surveillance and care.

## Materials and methods

### Participants

The birth cohort included 13,801 pregnant women who received antenatal care and delivery in the first affiliated Hospital of Anhui Medical University from January 2011 to December 2014^43^. The diagnosis of ICP was based on the presence of pruritus in combination with elevated serum levels of total bile acid (TBA ≥ 10 μmol/L). The study analyzed a total of 12,200 pregnant women, following the exclusion of 897 who withdrew or had no detailed delivery records, and the omission of a further 704 who had no diagnostic records of ICP. Biochemical parameters (aspartate transaminase, alanine transaminase, and bilirubin) were retrieved from the hospital records. The study was approved by the ethics committee of Anhui Medical University (Approval No. 20160010). A written informed consent was obtained from all participants, and all protocols were carried out in accordance with the approved guidelines.

### Measurement of serum TBA

Serum TBA levels were measured by an automatic biochemical analyzer (Dirui CS-T300, Ltd, Changchun, China) according to our previous protocol^44^.

### Statistical analysis

The data were analyzed using SPSS 20.0. Normal distribution of variables was assessed with the Shapiro–Wilk test. The mean differences were compared using non-parametric statistics (Mann–Whitney U test). Chi-square test (χ^2^ test) was used to compare categorical variables or ordinal variables. Crude and adjusted relative risks (RRs) of ICP with 95% confidence intervals (95% CI) were calculated using multiple logistic regression models. A *p* value of < 0.05 (two-tailed) or a 95% CI not including 1 and 0 (for relative risk) was considered to be statistically significant.
